# *NUDT15* polymorphism and *NT5C2* and *PRPS1* mutations influence thiopurine sensitivity in acute lymphoblastic leukaemia cells

**DOI:** 10.1111/jcmm.16981

**Published:** 2021-10-12

**Authors:** Shinpei Somazu, Yoichi Tanaka, Minori Tamai, Atsushi Watanabe, Keiko Kagami, Masako Abe, Daisuke Harama, Tamao Shinohara, Koshi Akahane, Kumiko Goi, Kanji Sugita, Takaya Moriyama, Jun Yang, Hiroaki Goto, Masayoshi Minegishi, Shotaro Iwamoto, Junko Takita, Takeshi Inukai

**Affiliations:** ^1^ Department of Pediatrics University of Yamanashi Yamanashi Japan; ^2^ Department of Clinical Pharmacy Center for Clinical Pharmacy and Sciences School of Pharmacy Kitasato University Minato‐ku Japan; ^3^ Department of Pharmaceutical Sciences St Jude Children's Research Hospital Memphis Tennessee USA; ^4^ Division of Hematology/Oncology Kanagawa Children's Medical Center Kanagawa Japan; ^5^ Tohoku Block Center Japanese Red Cross Society Sendai Japan; ^6^ Department of Pediatrics Mie University Graduate School of Medicine Tsu Japan; ^7^ Department of Pediatrics Kyoto University Kyoto Japan

**Keywords:** acute lymphoblastic leukemia, maintenance therapy, mercaputopurine, pharmacogenetics

## Abstract

In chemotherapy for childhood acute lymphoblastic leukaemia (ALL), maintenance therapy consisting of oral daily mercaptopurine and weekly methotrexate is important. *NUDT15* variant genotype is reportedly highly associated with severe myelosuppression during maintenance therapy, particularly in Asian and Hispanic populations. It has also been demonstrated that acquired somatic mutations of the *NT5C2* and *PRPS1* genes, which are involved in thiopurine metabolism, are detectable in a portion of relapsed childhood ALL. To directly confirm the significance of the *NUDT15* variant genotype and *NT5C2* and *PRPS1* mutations in thiopurine sensitivity of leukaemia cells in the intrinsic genes, we investigated 84 B‐cell precursor‐ALL (BCP‐ALL) cell lines. Three and 14 cell lines had homozygous and heterozygous variant diplotypes of the *NUDT15* gene, respectively, while 4 and 2 cell lines that were exclusively established from the samples at relapse had the *NT5C2* and *PRPS1* mutations, respectively. Both *NUDT15* variant genotype and *NT5C2* and *PRPS1* mutations were significantly associated with DNA‐incorporated thioguanine levels after exposure to thioguanine at therapeutic concentration. Considering the continuous exposure during the maintenance therapy, we evaluated in vitro mercaptopurine sensitivity after 7‐day exposure. Mercaptopurine concentrations lethal to 50% of the leukaemia cells were comparable to therapeutic serum concentration of mercaptopurine. Both *NUDT15* variant genotype and *NT5C2* and *PRPS1* mutations were significantly associated with mercaptopurine sensitivity in 83 BCP‐ALL and 23 T‐ALL cell lines. The present study provides direct evidence to support the general principle showing that both inherited genotype and somatically acquired mutation are crucially implicated in the drug sensitivity of leukaemia cells.

## 1 INTRODUCTION

Thiopurines such as mercaptopurine (6MP) and thioguanine (6TG) are widely used anticancer agents. In contemporary regimens for childhood acute lymphoblastic leukaemia (ALL), maintenance therapy consisting of oral daily 6MP and weekly methotrexate is an important component.^1^, ^2^ Its importance is directly indicated by the clinical observations showing that lower adherence to therapy increases relapse risk^3^ and shorter therapy increases relapse risk in low‐risk groups.^4^ Continuous exposure to 6MP during the maintenance therapy induces severe myelosuppression in some ALL patients. Genome‐wide association studies revealed a contribution of missense variant in the *NUDT15* gene to thiopurine‐induced myelosuppression in Asian and Hispanic patients.^5^, ^6^ In individuals with homozygous or compound heterozygous variant genotype of the *NUDT15* gene, tolerated dosages are significantly decreased.^5^, ^6^, ^7^, ^8^

NUDT15 is a nucleotide diphosphatase; it converts active thioguanine triphosphate (TGTP) to inactive thioguanine monophosphate (TGMP) in thiopurine metabolism.^7^ Accordingly, NUDT15 prevents incorporation of TGTP and deoxythioguanosine triphosphate (TdGTP) into DNA and thus negatively affects DNA damage‐induced cytotoxic effects of thiopurine.^7^ Since the majority of germline genetic variants are represented in leukaemia cells,^9^ inherited variants of *NUDT15* may influence the thiopurine sensitivity of leukaemia cells.^10^ However, the significance of the intrinsic variant genotype of *NUDT15* in thiopurine sensitivity of ALL cells is not fully elucidated in vitro in the human model.

In addition to the inherited genetic variants, acquired somatic mutations may also influence the thiopurine sensitivity of leukaemia cells. Recent studies in relapsed childhood ALL revealed that relapse‐specific somatic mutations were detectable in the genes involved in thiopurine metabolism such as *NT5C2* ^11^, ^12^, ^13^ and *PRPS1*.^14^, ^15^ NT5C2 is a nucleotidase involving in dephosphorylation of thiopurine‐generated 6‐hydroxypurine monophosphates.^16^, ^17^ *NT5C2* mutations reduce the cytotoxic effect of thiopurine due to a degradation of critical intermediate metabolites of thiopurine. PRPS1 is an enzyme essential for purine biosynthesis.^14^ *PRPS1* mutations impair thiopurine metabolism due to a defect in the purine salvage pathway. In previous reports, the significances of *NT5C2* and *PRPS1* mutations in thiopurine sensitivity were confirmed using ALL cell lines that were transduced with wild‐type or mutated cDNA by lentivirus vector.^11^, ^12^, ^14^, ^16^ Thus, the significances of *NT5C2* and *PRPS1* mutations in thiopurine sensitivity of ALL cells remain to be directly confirmed in the intrinsic genes.

Previous studies demonstrated that in vitro drug sensitivity profiles of primary leukaemia cells were significantly associated with clinical outcome in childhood ALL.^18^, ^19^ As an indicator of in vitro drug sensitivity of leukaemia cells, the 50% inhibitory concentration (IC50) value, which is drug concentration lethal to 50% of the leukaemia cells, is widely used.^18^, ^19^, ^20^ In the standard in vitro drug sensitivity assay, leukaemia cells are exposed to the agent for 2 to 4 days, and then, cell viabilities are evaluated with MTT or its derivative assays.^20^ In the present study, we investigated the significance of variant *NUDT15* genotype and *NT5C2* and *PRPS1* mutations in thiopurine activation and sensitivity of leukaemia cells using a large series of B‐cell precursor‐ALL (BCP‐ALL) and T‐cell ALL (T‐ALL) cell lines. We found that both variant *NUDT15* genotype and *NT5C2* and *PRPS1* mutations were significantly associated with the activation of thiopurine. However, neither variant *NUDT15* genotype nor *NT5C2* and *PRPS1* mutations were significantly associated with the sensitivities to thiopurine in the standard 3‐day incubation assay. As 6MP is administrated daily in maintenance therapy, we evaluated in vitro 6MP sensitivity after contentious exposure for 7 days, and found that both variant *NUDT15* genotype and *NT5C2* and *PRPS1* mutations were significantly associated with 6MP sensitivity.

## 2 MATERIALS AND METHODS

### 2.1 Cell lines

We used 84 BCP‐ALL cell lines (Table S1). Seventy‐seven cell lines were established from Japanese patients, while 7 cell lines were established from non‐Japanese patients. KOPN, KOCL, YAMN and YACL series of cell lines were sequentially established in our laboratory from 1980 to 2012.^21^, ^22^, ^23^ YCUB and KCB series of cell lines were sequentially established at Yokohama City University and Kanagawa Children's Medical Center^24^ and provided in 2014 (Dr. H. Goto). THP series of cell lines, L‐KUM and L‐ASK were sequentially established at Tohoku University^25^ and provided in 2014 (Dr. M. Minegishi). MB series of cell lines were sequentially established at Mie University Graduate School of Medicine^26^ and provided in 2014 (Dr. S. Iwamoto). Kasumi series of cell lines were sequentially established at Hiroshima University^27^ and provided in 2010 (Dr. T. Inaba). HBL‐3^28^ was established at Fukushima Medical University and provided in 2015 (Dr. H. Hojo). SU‐Ph2^29^ was established at Kindai University Faculty of Medicine and provided in 2010 (Dr. Y. Maeda). TCCY^30^ was established at Tochigi Cancer Center and provided in 2011 (Dr. Y. Sato). HALO1^21^ was provided in 1997 (Dr. A. T. Look at Dana‐Farber Cancer Institute). SK9^31^ was established at Tokyo Medical University and provided in 2012 (Dr. S. Okabe). Endokun^21^ was established at Iwate Medical University and provided in 1997 (Dr. M. Endo). SCMCL1 and SCMCL2^32^ were established at Saitama Children's Medical Center and provided in 2014 (Dr. J. Takita). P30/OHK^33^ and Nalm27^34^ were purchased from ATCC in 2012. We also used 23 T‐ALL cell lines (Table S2). KOPT‐K1 and KOPT‐5 were established in our laboratory. L‐MAT and L‐KAW were provided in 2014 (Dr. M. Minegishi). The other cell lines were provided in 2015 (Dr. A. T. Look in Dana‐Farber Cancer Institute, Boston, MA). All cell lines were maintained in RPMI‐1640 medium supplemented with 10% foetal calf serum in a humidified atmosphere of 5% CO_2_ at 37°C.

### 2.2 *NUDT15*and*TPMT*genotyping

After obtaining approval from the ethics committee of the University of Yamanashi, *NUDT15* genotype was analysed in the 84 BCP‐ALL cell lines and 23 T‐ALL cell lines. Coding regions (exons 1 and 3) of the *NUDT15* gene were first amplified by polymerase chain reaction (PCR) from germline DNA using the identical primers described in a previous report (Figure S1).^7^ Genotype was determined by Sanger sequencing of the PCR products. *TPMT* genotype of rs1142345, a representative variant SNP of the *TPMT* gene,^35^ was analysed in genomic DNA of 107 ALL cell lines by TaqMan SNP genotype assay (C_19567_20, Thermo Fisher Scientific, Waltham, MA).

### 2.3 *NT5C2*and*PRPS1* sequencing

Three regions of the *NT5C2* gene, including all of the previously identified mutation hotspots,^11^, ^12^, ^13^, ^16^, ^17^ were directly sequenced using following PCR products. Exon 2 was evaluated by sequencing genomic PCR products using forward (5′‐AAGATGGGGACCTCTTCATGTC‐3′) and reverse (5′‐TGTCTTTTCCCTTGGCTCCC‐3′) primers. Exons 8 – 15 were evaluated with following two RT‐PCR products: exons 7 – 13 were amplified using forward (5′‐AAGATGGGGACCTCTTCATGTC‐3′) and reverse (5′‐TGTCTTTTCCCTTGGCTCCC‐3′) primers and exons 11–16 were amplified using forward (5′‐GGATGCACGGAAACCACTCT‐3′) and reverse (5′‐ AACGCATCACTTGACTGGCA‐3′) primers. Whole coding region of the *PRPS1* gene was directly sequenced using two RT‐PCR products. Exons 1–4 were amplified using forward (5′‐GCGGAGTAGCAACGCAAAG‐3′) and reverse (5′‐TAGTGCAGTTCCTCCACTCAG‐3′) primers. Exons 4–7 were amplified using forward (5′‐CTGAGTGGAGGAACTGCACTAT‐3′) and reverse (5′‐TACCAAGGGGAAACAAGGGTG‐3′) primers. We also performed the target‐exon sequence analysis of the *NT5C2* and *PRPS1* genes (Ion AmpliSeq™ Designer v7.4.6.5, Thermo Fisher Scientific). The library was constructed using an Ion AmpliSeq Library Kit v2.0 and Ion Xpress Barcode Adaptors Kit (Thermo Fisher Scientific). After Agencourt AMPure XP purification (Beckman Coulter), individual libraries were amplified. The libraries were then processed with an Ion Chef System using an Ion PG Hi‐Q Chef Kit (Thermo Fisher Scientific). Next‐generation sequencing (NGS) was performed by using an Ion PGM Hi‐Q Sequencing Kit (Thermo Fisher Scientific) and 850 flows on an Ion 318 Chip Kit v2 (Thermo Fisher Scientific). After sequencing, single processing and base calling were performed using Torrent Suite 5.0.2 (Thermo Fisher Scientific). Output data were analysed using the Ion Reporter™ 5.16.0.2. Variant Call Format (VCF) files, then, were converted to Mutation Annotation Format (MAF) files using vcf2maf utility (https://github.com/mskcc/vcf2maf).

### 2.4 Quantification of cellular DNA‐TG level

To determine thiopurine metabolism in vitro, cells were treated with 0.1 µM of 6TG for 48 h. DNA‐incorporated thioguanine (DNA‐TG) levels were analysed with liquid chromatography‐tandem mass spectrometry (LC‐MS/MS) as described previously^36^ using 1 µl of 36.8 µM 6‐methylmercaptopurine‐d3 (Toronto Research Chemicals) as an internal standard. In brief, 100 µl of DNA extracted from the cells was denatured at 100°C for 5 min and, then, kept on ice for 2 min. The denatured DNA was digested by 0.6 U nuclease P1 from Penicillium citrinum (Sigma‐Aldrich) with10 µl of 10× digestive buffer (500 mM sodium acetate, 10 mM MgCl_2_ pH 5.3) at 50°C for 60 min. The digested DNA was treated with 3 U of calf intestinal alkaline phosphatase in the presence of 10× reaction buffer (Toyobo) at 37°C for 30 min and measured by LC‐MS/MS (ACQUITY H‐class and Xevo TQD, Waters). TG concentration was adjusted according to the amount of DNA.

### 2.5 AlamarBlue cell viability assay and detection of apoptosis

To determine the IC50 of 6MP and 6TG, an alamarBlue cell viability assay (Bio‐Rad Laboratory) was performed,^22^, ^37^ In the 3‐day incubation assay, a total of 1–4 × 10^5^ cells were plated into each well of a 96‐well flat‐bottom plate in triplicate in the absence or presence of seven different concentrations of 6MP (1.8–1,314 µM) and 6TG (0.25–180 µM). Cells were cultured for 68 h without washing the cells, and then, alamarBlue was added. In the 7‐day incubation assay, after initial culture for 3 days in the absence or presence of seven different concentrations of 6MP (0.012–3 µM), equal volumes of fresh medium containing the same concentrations of 6MP were added into each corresponding well. Each cell line was cultured for a total of 7 days, and then, alamarBlue was added. To determine the IC50 values of representative agents, each cell line was incubated for 2 days (DNR, daunorubicin; VCR, vincristine; AraC, cytarabine; Maf, mafosfamide) or 3 days (Dex, dexamethasone; Pred, prednisolone; L‐Asp, l‐asparaginase; MTX, methotrexate).^38^ After a 6‐h additional incubation with alamarBlue, the absorbance at 570 nm was monitored by a microplate spectrophotometer using 600 nm as a reference wavelength. Cell viability was calculated by expressing the ratio of the optical density of the treated wells to that of the untreated wells as a percentage. The concentration of agent required to reduce the viability of the treated cells to 50% of the untreated cells was calculated by curve fitting with polynomial approximation, and the median of the values measured by three independent assays was determined as the IC50. To detect apoptosis, five representative BCP‐ALL cell lines were cultured in the absence or presence of 20 µM of 6MP for 3 days and in the absence or presence of 0.2 µM of 6MP for 7 days (after 3 days culture, equal volumes of fresh medium containing 0.2 µM of 6MP were added). The cells were stained with a fluorescein isothiocyanate (FITC)‐conjugated Annexin‐V (MBL). The stained cells were analysed by flow cytometry (BD FACSCalibur, BD Biosciences).

### 2.6 Statistics

Correlation coefficient was evaluated using Excel software (version 16.16.27). In each evaluation, median value was compared. Mann‐Whitney test, Fisher's exact test and Pearson's correlation analysis were performed using R (version 3.5.2) software.

## 3 RESULTS

### 3.1 Thiopurine sensitivities in standard 3‐day incubation assay

**FIGURE 1:**
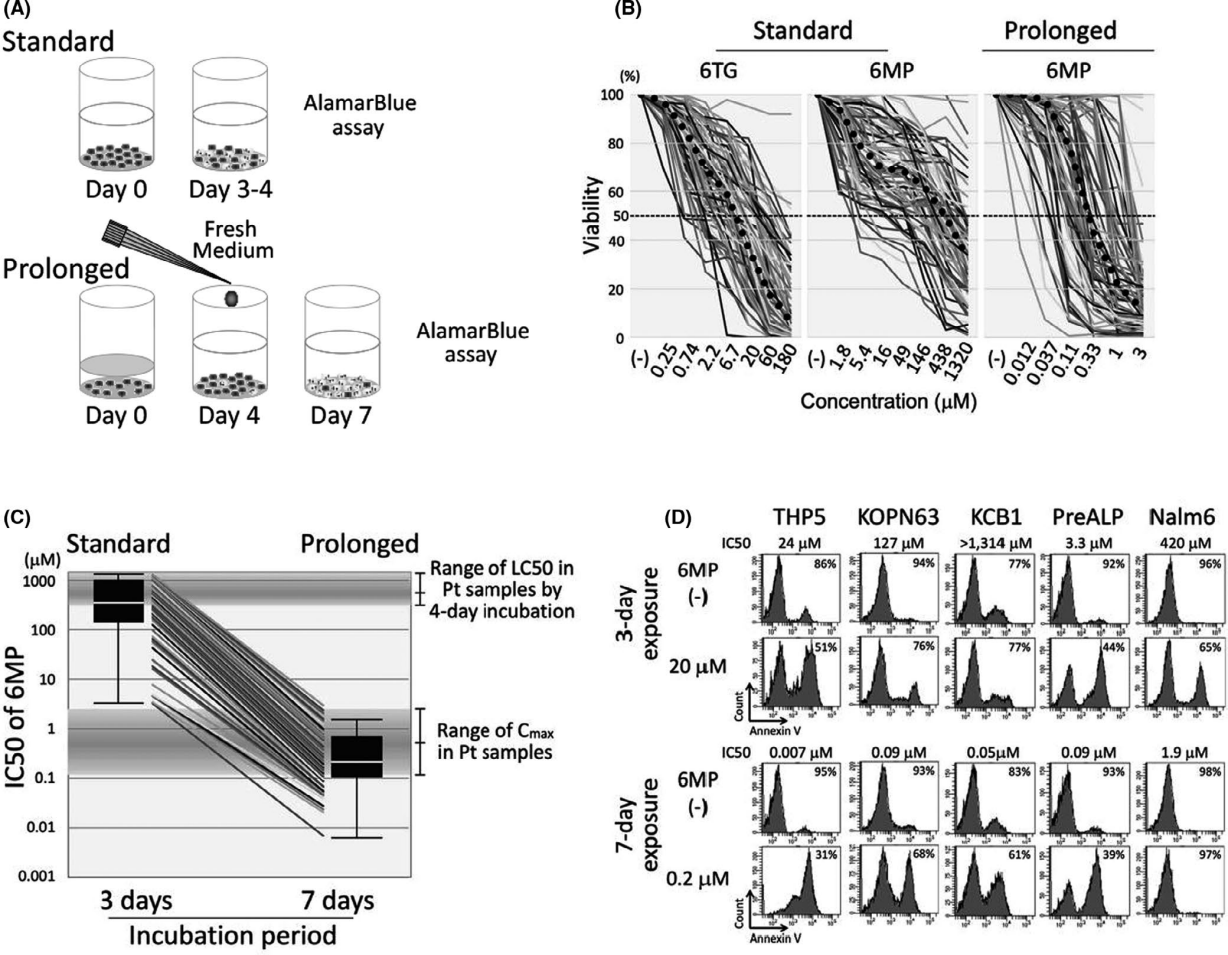
In vitro thiopurine sensitivities of BCP‐ALL cell lines in standard 3‐day and prolonged 7‐day incubation assays. (A) Schematic representation of the standard 3‐day incubation assay (bottom panel) and the prolonged 7‐day incubation assay (bottom panel). In the 7‐day incubation assay, fresh medium containing 6MP was added to culture medium, and the cells were cultured until day 7 in the presence of 6MP. (B) Dose‐response curves of thiopurine sensitivities in BCP‐ALL cell lines. Horizontal and vertical axes indicate log concentration of thiopurine and cell viability, respectively. Left, middle, and right panels indicate dose‐response curves of 6TG in the standard 3‐day incubation assay, 6MP in the standard 3‐day incubation assay and 6MP in the prolonged 7‐day incubation assay, respectively. Bold dotted lines indicate dose‐response curves of median values. (C) Comparison of IC50 value of 6MP in each cell line after 3‐day exposure (left) and 7‐day exposure (right). Vertical axis indicates log concentration of 6MP. Top and middle boxes indicate previously reported the IC50 value range of patients’ samples and that of serum Cmax values in the patients during maintenance therapy, respectively. (D) Induction of apoptosis in five representative BCP‐ALL cell lines treated with 20 µM of 6MP for 3 days (upper panels) and in those treated with 0.2 µM of 6MP for 7 days (lower panels). IC50 values in alamarBlue assay and percentages of viable (negative for Annexin V‐binding) cells are indicated in each panel

We evaluated thiopurine sensitivities of BCP‐ALL cell lines by alamarBlue assay after a 3‐day incubation period (Figure 1A). In the majority of cell lines, the dose‐response curve of 6MP showed a biphasic sigmoid pattern (Figure 1B). The median IC50 (day 3) value of 6MP (410 µM) and 6TG (9.8 µM) in the 84 cell lines was almost similar to previously reported IC50 values in primary childhood ALL samples determined by an MTT assay after a 4‐day incubation period (Figure 1C). ^7^, ^20^, ^39^However, the median IC50 (day 3) value of 6MP was approximately 800 times higher than previously reported C_max_ value of 6MP (median; 0.56 µM, range; 0.13–2.3 µM) during daily oral administration of standard dose of 6MP in childhood ALL patients.^40^ Moreover, the median IC50 (day 3) value of 6TG was approximately 30 times higher than previously reported C_max_ values of 6TG (0.31 µM, 0.05–0.74 µM) during daily oral administration of standard dose of 6TG in childhood ALL patients.^41^ These marked discrepancies between in vitro IC50 values and therapeutic serum concentrations suggested that standard 3–4 day incubation assay in vitro might be inadequate for evaluating anti‐leukemic activity of thiopurine during the maintenance therapy.

### 3.2 6MP sensitivity in continuous 7‐day incubation assay

Considering that 6MP is daily administrated in maintenance therapy, we evaluated 6MP sensitivity of BCP‐ALL cell lines after a prolonged continuous exposure for 7 days (Figure 1A). The dose‐response curve of 6MP showed a monophasic sigmoid pattern (Figure 1B). Of note, median IC50 (day 7) value of 6MP (0.25 µM) in BCP‐ALL cell lines was approximately 1,600 times lower than the median IC50 (day3) value of 6MP (410 µM) (Figure 1C). Moreover, the IC50 (day 7) value range of 6MP in BCP‐ALL cell lines was similar to the serum C_max_ level of 6MP during maintenance therapy.^40^ Among six representative fusion genes, IC50 (day 7) value in 14 cell lines with *MLL* fusion was significantly higher than that in 16 cell lines with *BCR*/*ABL1* or *BCR*/*ABL1*‐like fusions (*p *= 0.048 in Mann‐Whitney test) and in 4 cell lines with *TCF3*/*HLF* fusion (*p *= 0.044) (Figure S2). Moreover, the IC50 (day 7) values in 46 BCP‐ALL cell lines established at relapse (median; 0.41 µM) were significantly higher than those in 29 BCP‐ALL cell lines established at diagnosis (0.15 µM) (*p *= 0.0104 in Mann‐Whitney test) (Figure S3).

Next, we evaluated induction of apoptosis in five representative cell lines (Figure 1D). After 3‐day exposure to 20 µM (approximately 40 times higher concentration than median serum C_max_ level) of 6MP, cell viability was decreased to less than 70% in three cell lines. In contrast, after 7‐day exposure to 0.2 µM (approximately half concentration of median serum C_max_ level) of 6MP, cell viability was decreased to less than 70% and 40% in four and two cell lines, respectively. These observations indicated that the prolonged continuous exposure to 6MP for 7 days is a reliable in vitro model system that may mimic the anti‐leukemic activity of 6MP during maintenance therapy.

### 3.3 Differences in anti‐leukemic properties of 3‐day and 7‐day exposures to 6MP

**FIGURE 2:**
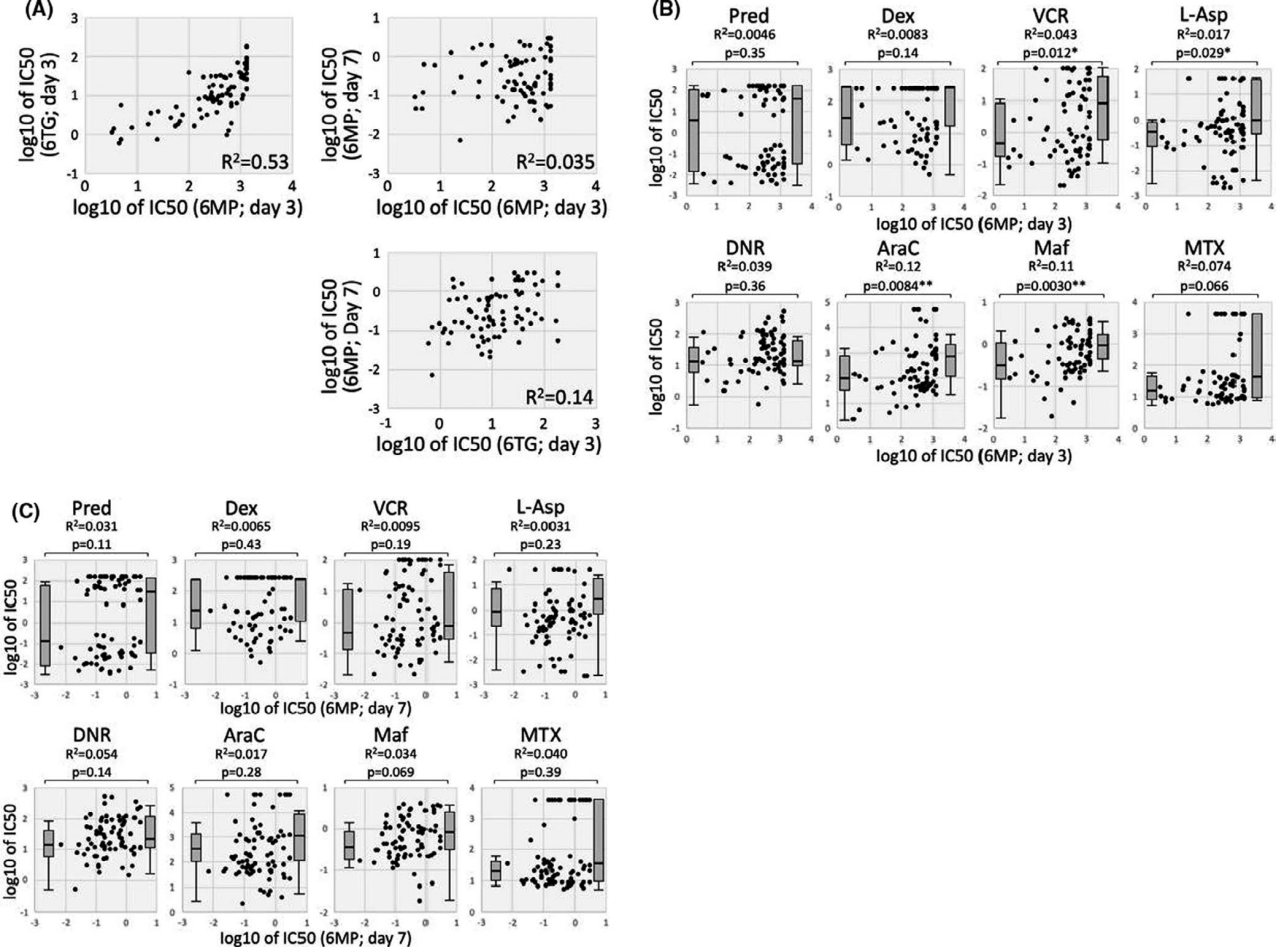
Differences in anti‐leukemic properties of 6MP in 3‐day and 7‐day exposures. (A) Correlation between the IC50 (day 3) values of thiopurine and the IC50 (day 7) value of 6MP in each cell line. Coefficient of correlation (R2) is indicated in each panel. (B and C) Associations of the 6MP sensitivities in 3‐day exposure (B) and in 7‐day exposure (C) with the sensitivities to eight representative chemotherapeutic agents. Horizontal and vertical axes indicate log IC50 value of 6MP and that of each agent, respectively. Coefficient of correlation (R2) is indicated in each panel. Left and right box plots indicate IC50 value of cell lines with lower IC50 value of 6MP than median value and that of cell lines with higher IC50 value of 6MP than median value, respectively. *p* value between two groups in Mann‐whiney analysis is indicated in each panel

We compared the anti‐leukemic properties of 6MP in BCP‐ALL cell lines during 3‐day exposure and 7‐day exposure. In the 3‐day incubation assay, a significant positive correlation was observed between the IC50 (day3) value of 6MP and that of 6TG (R^2 ^= 0.55, *p *< 0.0001; Figure 2A). However, the IC50 (day 7) value of 6MP did not show a significant correlation with either the IC50 (day3) value of 6MP or that of 6TG (Figure 2A). Then, we evaluated associations of 6MP sensitivity with the sensitivities to eight representative chemotherapeutic agents. In the 3‐day incubation assay (Figure 2B), the cell lines with higher IC50 (day 3) values of 6MP than the median value were significantly more resistant to VCR, L‐Asp, AraC and CY (Maf) than the cell lines with lower IC50 (day 3) values of 6MP. In contrast, in the 7‐day incubation assay (Figure 2C), no significant differences were observed in the sensitivities to eight agents between the cell lines with higher IC50 (day 7) values of 6MP and those with lower IC50 (day 7) values of 6MP (Figure 2C). These observations indicated that continuous exposure to pharmacological concentration of 6MP may exert unique anti‐leukemic properties without cross resistance to the other representative chemotherapeutic agents.

### 3.4 Significance of *NUDT15* genotype in DNA‐incorporated thioguanine level

**FIGURE 3:**
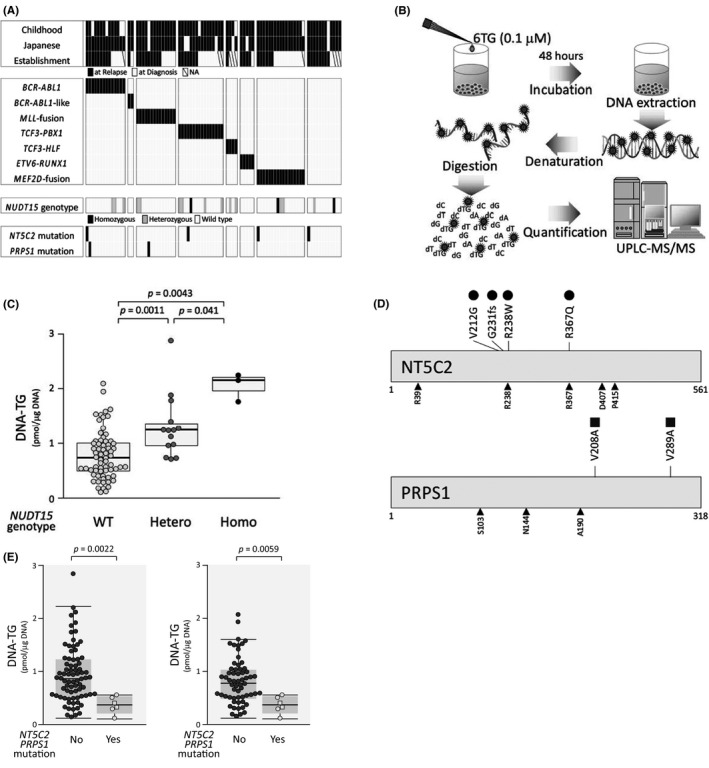
Significance of *NUDT15* genotype and *NT5C2* and *PRPS1* mutations in DNA‐incorporated thioguanine level. (A) Heat map of information for cell line establishment and genetic features, with characteristics in rows and cell lines in columns. (B) Schematic representation of experimental flow in the measurement of cellular thioguanine level incorporated into DNA (DNA‐TG). (C) Association of *NUDT15* genotype with DNA‐TG level in the 84 BCP‐ALL cell lines incubated with 0.1 µM of 6TG for 48 h. *p*‐value in Mann‐whiney analysis is indicated. (D) Locations of mutations in the *NT5C2* gene (top panel) and in the *PRPS1* gene (bottom panel). Closed circles and closed rectangle indicate amino acid position of *NT5C2* and *PRPS1* mutations, respectively, and arrow heads indicate previously reported amino acid position of the hotspot mutations in BCP‐ALL patients’ samples. (E) Association of *NT5C2* (open circles) and *PRPS1* (open rectangle) mutations 4 with DNA‐TG level in 84 BCP‐ALL cell lines (left) and in 67 cell lines with wild‐type *NUDT15* genotype (right) incubated with 0.1 µM of 6TG for 48 h. *p*‐value in Mann‐Whitney analysis is indicated

*NUDT15* genotype has been reported to be highly associated with susceptibility to thiopurine‐induced bone marrow suppression, particularly in Asian populations.^7^ We determined *NUDT15* genotype by directly sequencing the genomic PCR products of exons 1 (Figure S4) and 3^7^ (Figure S5) in the 84 BCP‐ALL cell lines (Supplemental Table). Sixty‐seven cell lines (79.8%) showed the wild‐type diplotype, while 14 (16.7%) and 3 (3.6%) cell lines showed the heterozygous and homozygous variant diplotypes, respectively (Figure 3A). All of the 17 cell lines with variant genotypes were established from Japanese patients (Figure 3A). We also evaluated genotype of rs1142345, a representative variant SNP of the *TPMT* gene,^35^ by TaqMan SNP genotype assay, but none of 84 BCP‐ALL cell lines had variant genotype (data not shown).

Then, we evaluated the level of thioguanine incorporated into cellular DNA (DNA‐TG) in each cell line 48 h after exposure to 0.1 µM (approximately 30% concentration of C_max_ value) of 6TG (Figure 3B). DNA‐TG levels in three homozygous variant cell lines (median; 2,147 fmol/µgDNA) were significantly higher than those in 14 heterozygous variant cell lines (1,247 fmol/µgDNA, *p *= 0.044) and those in 67 wild‐type cell lines (741 fmol/µgDNA, *p *= 0.0043) (Figure 3C). Moreover, DNA‐TG levels in 14 heterozygous variant cell lines were significantly higher than those in 67 wild‐type cell lines (*p *= 0.0011). These observations demonstrated that *NUDT15* genotype is strongly associated with thiopurine metabolism in BCP‐ALL cell lines.

### 3.5 Significance of *NT5C2* and *PRPS1* mutations in DNA‐incorporated thioguanine level

Mutations in the *NT5C2* ^11^, ^12^, ^13^, ^15^ and *PRPS1* ^14^, ^15^ genes are reportedly associated with thiopurine resistance in the relapsed ALL cases. Therefore, we investigated *NT5C2* (located on 10q24.32‐q24.33) and *PRPS1* (Xq22.3) mutations in 84 BCP‐ALL cell lines (Figure 3D) (Table S1). Heterozygous mutations of the *NT5C2* gene were observed in four cell lines (Figure S6), and hemizygous mutation of the *PRPS1* gene was observed in one cell line (Figure S7) by direct sequences of the RT‐PCR products. In the target‐exon sequence analysis with NGS, the identical mutations were observed in the five cell lines (Table S3). Additionally, the *PRPS1* mutation was detectable in RS4;11.

Of note, all of the six cell lines with mutations were established from the samples at relapse (6/47 cell lines; 12.8%), while no mutations were observed in the cell lines established from the samples at diagnosis (0/29 cell lines) (*p *= 0.077 in Fisher's exact test) (Figure 3A). We next evaluated an association of *NT5C2* and *PRPS1* mutations with DNA‐TG levels in BCP‐ALL cell lines treated with 6TG. Of note, DNA‐TG levels in six cell lines with *NT5C2* and *PRPS1* mutations (median; 366 fmol/µgDNA) were significantly lower than 78 cell lines without mutations (894 fmol/µgDNA, *p *= 0.0022) (Figure 3E). We focused on 67 BCP‐ALL cell lines with the wild‐type *NUDT15* genotype. DNA‐TG levels in six cell lines with *NT5C2* and *PRPS1* mutations (366 fmol/µgDNA) were significantly lower than the 61 cell lines without mutations (820 fmol/µgDNA, *p *= 0.0059) (Figure 3E). These observations indicated that *NT5C2* and *PRPS1* mutations are strongly associated with a decreased incorporation of thioguanine into cellular DNA, independent of the *NUDT15* genotype in BCP‐ALL cell lines.

### 3.6 Significance of *NUDT15* genotype and *NT5C2* and *PRPS1* mutations in prolonged 7‐day incubation assay

**FIGURE 4:**
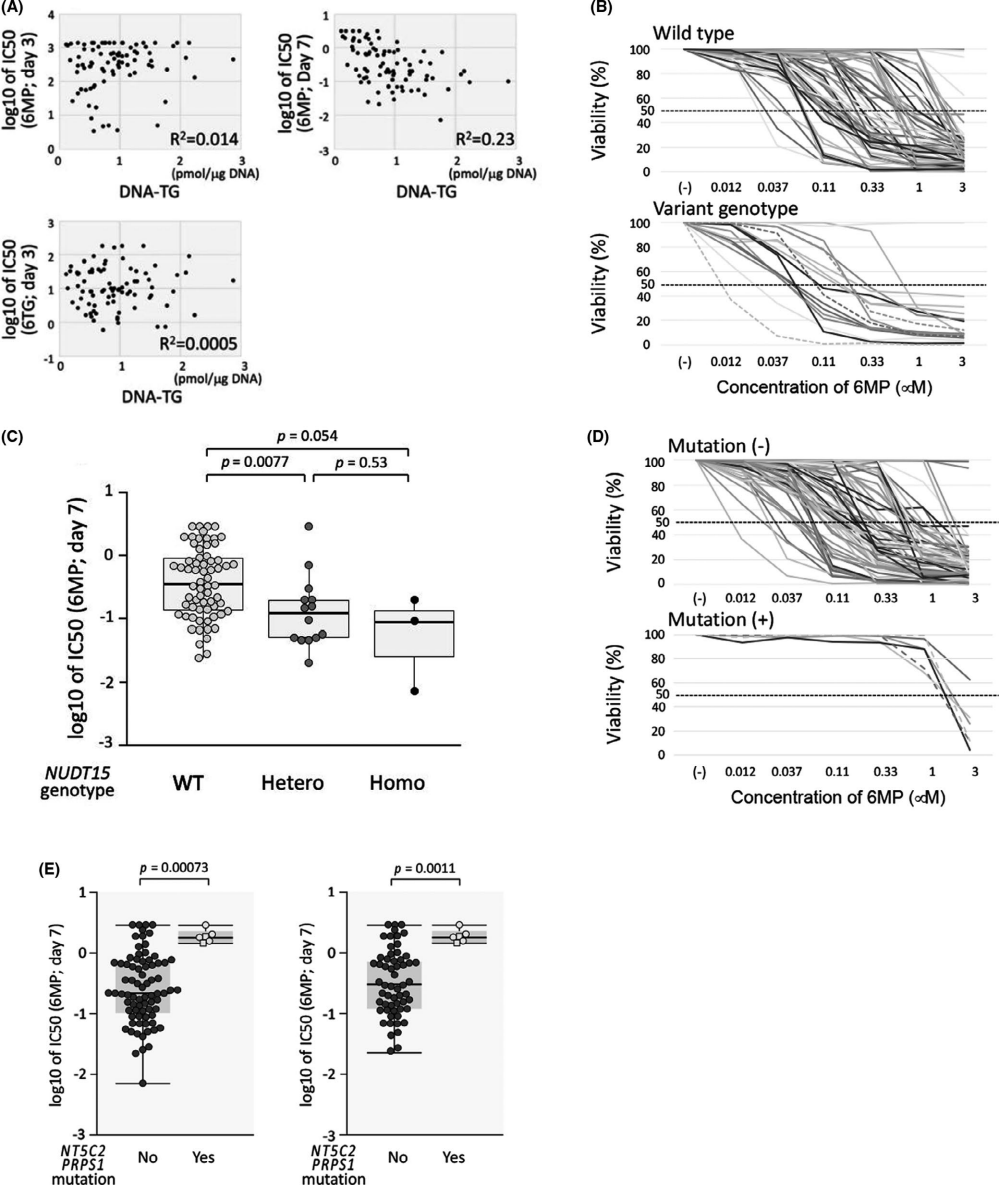
Significance of *NUDT15* genotype and *NT5C2* and *PRPS1* mutations in thiopurine sensitivity. (A) Correlation of thiopurine sensitivity with cellular thioguanine level incorporated into DNA (DNA‐TG) in the 84 BCP‐ALL cell lines incubated with 0.1 µM of 6TG for 48 h. Horizontal and vertical axes indicate log IC50 value of thiopurine in 3‐day and 7‐day incubation and level of DNA‐TG, respectively. Coefficient of correlation (R2) is indicated in each panel. (B) Dose‐response curves of 6MP sensitivity in prolonged 7‐day incubation assay. Top and bottom panels indicate dose‐response curves of cell lines with wild‐type *NUDT15* genotype and those of cell lines with variant *NUDT15* genotype, respectively. In the bottom panel, dotted lines indicate dose‐response curves of cell lines with homozygous variant genotype. (C) Association of *NUDT15* genotype with log IC50 (day 7) values of 6MP in the 83 BCP‐ALL cell lines. *p*‐value in Mann‐Whitney analysis is indicated. (D) Dose‐response curves of 6MP sensitivity in the prolonged 7‐day incubation assay. Top and bottom panels indicate dose‐response curves of cell lines without *NT5C2* and *PRPS1* mutations and those of cell lines with mutations, respectively. In the bottom panel, dotted lines indicate dose‐response curves of the cell lines with *PRPS1* mutation. (E) Association of *NT5C2* and *PRPS1* mutations with the log IC50 (day 7) values of 6MP in the 83 BCP‐ALL cell lines (left) and in the 67 cell lines with wild‐type *NUDT15* genotype (right). *p*‐value in Mann‐Whitney analysis is indicated

We evaluated the significance of *NUDT15* genotype and *NT5C2* and *PRPS1* mutations in thiopurine sensitivities. Prior to the evaluation, we verified whether DNA‐TG levels in BCP‐ALL cell lines were associated with thiopurine sensitivities (Figure 4A). In the 3‐day incubation assay, DNA‐TG levels of the cell lines treated with 6TG did not show a significant correlation with either IC50 (day 3) values of 6MP or those of 6TG in BCP‐ALL cell lines. Moreover, IC50 (day 3) values of 6MP and 6TG in BCP‐ALL cell lines were not significantly associated with either *NUDT15* variant genotype (Figure S8) or *NT5C2* and *PRPS1* mutations (Figure S9). In contrast to the 3‐day incubation assay, the intra‐cellular DNA‐TG levels showed a significant negative correlation with IC50 (day 7) values of 6MP (R^2 ^= 0.23, *p *< 0.001; Figure 4A). Then, we investigated the association of *NUDT15* variant genotype with 6MP sensitivity in the prolonged 7‐day incubation assay (Figure 4B and C). Of note, the IC50 (day 7) value of 6MP was significantly associated with *NUDT15* genotype. Sixty‐seven cell lines with wild‐type genotype (median IC50; 0.36 µM) were significantly more resistant than the 14 cell lines with heterozygous variant genotype (0.12 µM, *p *= 0.0077) and tended to be more resistant than the 3 cell lines with homozygous variant genotype (0.09 µM, *p *= 0.055). Moreover, among 46 cell lines established at relapse, variant *NUDT15* genotype tended to be associated with better 6MP sensitivity (Figure S10).

Next, we evaluated the significance of *NT5C2* and *PRPS1* mutations in 6MP sensitivity in the prolonged 7‐day incubation assay (Figure 4D). Of note, six cell lines with *NT5C2* and *PRPS1* mutations (median IC50; 1.85 µM) were significantly more resistant to 6MP than 77 cell lines without mutations (0.22 µM, *p *= 0.00073) (Figure 4E). In 46 BCP‐ALL cell lines established at relapse, *NT5C2* and *PRPS1* mutations were significantly associated with 6MP resistance (Figure S10). In 67 BCP‐ALL cell lines with the wild‐type *NUDT15* genotype, the IC50 (day 7) value in six cell lines with *NT5C2* and *PRPS1* mutations was significantly higher than 61 cell lines without mutations (0.32 µM, *p *= 0.0011) (Figure 4E). These observations demonstrated that both *NUDT15* variant genotype and *NT5C2* and *PRPS1* mutations are independently associated with 6MP sensitivity in BCP‐ALL cell lines.

### 3.7 Significance of *NUDT15* genotype and *NT5C2* and *PRPS1* mutations in T‐ALL cell lines

**FIGURE 5:**
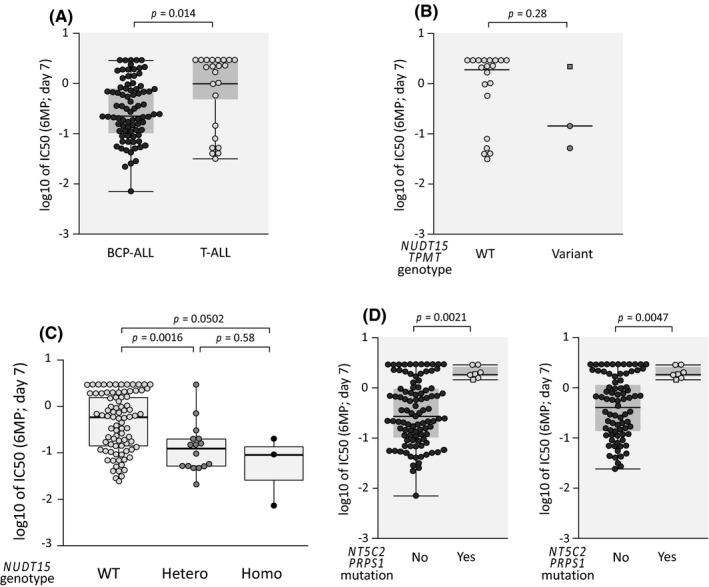
Significance of *NUDT15* and *TPMT* genotypes and *NT5C2* and *PRPS1* mutations in mercaptopurine sensitivity of ALL cell lines including T‐ALL in prolonged 7‐day incubation assays (A) Comparison of 6MP sensitivities between 83 BCP‐ALL cell lines and 23 T‐ALL cell lines.(B) Association of *NUDT15* and *TPMT* genotypes with log IC50 (day 7) values of 6MP in the 23 BCP‐ALL cell lines. Bars indicate median values. Circle and square indicate cell lines with *NUDT15* and *TPMT* variant genotypes, respectively. (C) Association of *NUDT15* genotype with log IC50 (day 7) values of 6MP in the 106 ALL (83 BCP‐ALL and 23 T‐ALL) cell lines. (D) Association of *NT5C2* and *PRPS1* mutations with the log IC50 (day 7) values of 6MP in106 ALL cell lines (left) and in the 88 ALL (67 BCP‐ALL and 21 T‐ALL cell lines) cell lines with wild‐type *NUDT15* genotype (right). In each Figure, *p*‐value in Mann‐Whitney analysis is indicated

In 23 T‐ALL cell lines (Table S2), we evaluated the variant genotype of the *NUDT15* and *TPMT* genes, and confirmed heterozygous variant genotypes of the *NUDT15* and *TPMT* genes in two and one cell lines, respectively. We also performed the exon sequencing analysis of the *NT5C2* and *PRPS1* genes, and confirmed heterozygous mutation of the *NT5C2* gene (R29Q, 60.2%) in one cell line (BE‐13) (Table S3). Then, we evaluated in vitro 6MP sensitivity in 7‐day incubation assay. Of note, 23 T‐ALL cell lines (median IC50; 2.0 µM) were significantly more resistant to 6MP than 83 BCP‐ALL cell lines (0.25 µM, *p *= 0.014 in Mann‐Whitney test) (Figure 5A). Although statistically insignificant, three cell lines with variant genotypes of the *NUDT15* and *TPMT* genes (median IC50; 0.13 µM) tended to be more sensitive to 6MP than 20 cell lines with wild‐type genotypes (2.2 µM, *p *= 0.28 in Mann‐Whitney test) (Figure 5B). In contrast, one cell line with the *NT5C2* mutation (BE‐13) was highly resistant to 6MP (IC50; >3 µM).

We finally evaluated IC50 (day 7) values in 106 ALL cell lines (83 BCP‐ALL cell lines and 23 T‐ALL cell lines). Sixty‐three cell lines established at relapse (median IC50; 0.69 µM) were significantly more resistant to 6MP than 35 cell lines established at diagnosis (0.16 µM, *p *= 0.0076 in Mann‐Whitney test) (Figure S11). The significant association was observed between the *NUDT15* variant genotype and higher 6MP in 106 cell lines (Figure 5C) and in 63 cell lines established at relapse (Figure S12). Moreover, the significant association of the *NT5C2* and *PRPS1* mutations with the 6MP resistance was observed in all of the 106 cell lines (Figure 5D), in 63 cell lines established at relapse (Figure S12) and in 88 cell lines (67 BCP‐ALL cell lines and 21 T‐ALL cell lines) with the wild‐type *NUDT15* genotype (Figure 5D). No clear association was observed between % mutated sequences and IC50 (day 7) values of 6MP in the 7 cell lines with the *NT5C2* or *PRPS1* mutations (Figure S13). These observations demonstrated that both *NUDT15* variant genotype and *NT5C2* and *PRPS1* mutations are independently associated with 6MP sensitivity in ALL cell lines.

## 4 DISCUSSION

In the present study, using a large panel of BCP‐ALL cell lines, we investigated the significance of both inherited variant genotype and somatically acquired mutation on thiopurine activation and sensitivity. First, we focused on the variant genotypes of the *NUDT15* gene. Among 77 Japanese BCP‐ALL cell lines, 14 (18.2%) and 3 (3.9%) cell lines had the heterozygous and homozygous variant diplotypes of the *NUDT15* gene, respectively. Regarding the rs116855232 genotype—the most common variant of the *NUDT15* gene^7^—13 (16.9%) and 3 (3.9%) cell lines had the heterozygous and homozygous variant haplotypes, respectively. Thus, the minor allele frequency (MAF) of rs116855232 in 77 Japanese BCP‐ALL cell lines was 0.123. In three previous studies of Japanese childhood ALL, MAF of rs116855232 was reportedly 0.1–0.163.^42^, ^43^, ^44^ Accordingly, the frequency of variant *NUDT15* genotypes in our Japanese BCP‐ALL cell lines was similar to that in Japanese childhood ALL patients. Using 84 BCP‐ALL cell lines that included 7 non‐Japanese cell lines, we confirmed the significant association of variant *NUDT15* genotype with higher intra‐cellular DNA‐TG levels after exposure to therapeutic concentration of 6TG. Our observation in BCP‐ALL cell lines was consistent with the previous clinical notion that variant genotype of the *NUDT15* gene was associated with higher DNA‐TG level in white blood cells from children with ALL receiving daily 6MP treatment.^7^

Next, we investigated somatic mutations of the *NT5C2* and *PRPS1* genes in BCP‐ALL cell lines. In the previous reports of childhood ALL, *NT5C2* and *PRPS1* mutations are exclusively observed in the samples at disease relapse.^11^, ^12^, ^13^, ^14^, ^15^ Consistent with these clinical notions, *NT5C2* and *PRPS1* mutations were exclusively observed in the cell lines established from the samples at relapse. Using these BCP‐ALL cell lines, we confirmed that intra‐cellular DNA‐TG levels (after exposure to a therapeutic concentration of 6TG for 2 days) were significantly lower in the cell lines with *NT5C2* and *PRPS1* mutations than in the cell lines without mutations. Thus, our observation in BCP‐ALL cell lines was consistent with the previous notion that these gain‐of‐function mutations of *NT5C2* ^11^, ^12^, ^16^, ^17^ and *PRPS1* ^13^ induce a reduction in active phosphorylated derivatives of thiopurine.

Under these circumstances, both variant genotype of *NUDT15* and mutations of *NT5C2* and *PRPS1* must be associated with in vitro thiopurine sensitivity of BCP‐ALL cell lines. To evaluate in vitro drug sensitivity of leukaemia cells, IC50 values determined by MTT or its derivative assays after 2–4 days exposures to the drug are widely used as a standard index.^20^ Thus, we first evaluated in vitro thiopurine sensitivity of BCP‐ALL cell lines after 3‐day exposure. Median IC50 (day 3) values of 6MP and 6TG in 84 BCP‐ALL cell lines were almost comparable to previously reported IC50 values of 6MP^39^ and 6TG^7^, ^20^, ^39^ in the patients’ samples, which were determined in 4‐day exposure assays. However, IC50 (day 3) values of 6MP and 6TG in BCP‐ALL cell lines were not significantly associated with either *NUDT15* genotype or *NT5C2* and *PRPS1* mutations. In this context, we noticed that the IC50 (day 3) values of 6MP and 6TG in our BCP‐ALL cell lines were approximately 800 and 30 times higher than previously reported serum C_max_ levels of 6MP^41^ and 6TG,^40^ respectively. Moreover, the assumed AUC for median IC50 (day 3) values of 6MP and 6TG were approximately 6,000 and 400 times higher than previously reported serum AUC values of 6MP^40^ and 6TG,^41^ respectively.

**FIGURE 6:**
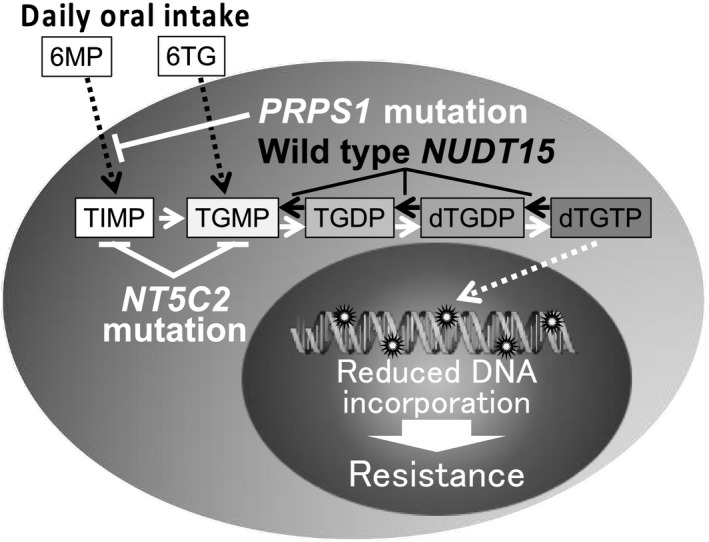
Significance of *NUDT15* genotype and *NT5C2* and *PRPS1* mutations in thiopurine sensitivity

In maintenance therapy for childhood ALL, 6MP is orally administrated every day. Thus, continuous exposure to therapeutic serum level of 6MP in vitro must be essential for a proper evaluation of anti‐leukemic activity of 6MP during the maintenance therapy. Accordingly, we investigated in vitro 6MP sensitivity after the continuous exposure for 7 days. Surprisingly, the median IC50 value of 6MP in the prolonged 7‐day assay was approximately 1,600 times lower than that in the standard 3‐day incubation assay, and no correlation was observed between IC50 (day 7) value and IC50 (day 3) value of 6MP. Of clinical importance, the range of IC50 values of 6MP in the prolonged 7‐day assay was comparable to the serum C_max_ level of 6MP during maintenance therapy.^35^ Moreover, the assumed AUC for median IC50 (day 7) value of 6MP was approximately four times higher than median AUC value of 6MP and was within the range of AUC value in the clinical setting.^40^ Consistently, it was reported that intra‐cellular 6MP metabolite levels of peripheral erythrocytes in the patients during maintenance therapy were increased gradually until day 3, subsequently, in an exponential way and reached a plateau around day 7.^45^ These observations suggested that continuous 7‐day exposure assay may be a practicable in vitro model that mimics anti‐leukemic activity of 6MP during the maintenance therapy. Indeed, both variant *NUDT15* genotype and *NT5C2* and *PRPS1* mutations were significantly associated with 6MP sensitivity of BCP‐ALL and T‐ALL cell lines in the prolonged 7‐day assay. When focused on the cell lines with the wild‐type *NUDT15* genotype, *NT5C2* and *PRPS1* mutations were significantly associated with 6MP resistance. These observations indicated that both variant genotype of *NUDT15* and mutations of *NT5C2* and *PRPS1* are independently associated with thiopurine sensitivity of BCP‐ALL and T‐ALL (Figure 6). This is the first direct confirmation that the intrinsic *NT5C2* and *PRPS1* mutations are associated with in vitro 6MP resistance as a result of reduced DNA‐TG level.

Even in recent intensified multi‐agent chemotherapeutic regimens for childhood ALL, maintenance therapy is an indispensable component.^1^, ^2^ The essential role of maintenance therapy suggests that continuous exposure to 6MP at pharmacological concentration (*eg* less than 1 µM) may be crucial for anti‐leukemic properties of 6MP during the maintenance therapy. To verify the unique anti‐leukemic properties of 6MP during the maintenance therapy, we evaluated the correlation between 6MP sensitivity and sensitivities to other chemotherapeutic agents in BCP‐ALL cell lines. However, no significant correlation was observed between 6MP sensitivity in the continuous 7‐day exposure and sensitivities to representative agents including glucocorticoids, vincristine, l‐asparaginase and daunorubicin. These observations suggested that the anti‐leukemic properties of 6MP in the maintenance therapy may not overlap with anti‐leukemic properties of other agents. In some regimens for childhood ALL, maintenance therapy is intensified with vincristine plus prednisolone or dexamethasone pulses.^2^, ^46^ Our observations in BCP‐ALL cell lines may support the pharmacological efficacy in using pulses of vincristine and glucocorticoids during the maintenance therapy.

In conclusion, 7‐day incubation assay of 6MP sensitivity is a reasonable in vitro system to evaluate the anti‐leukemic properties of 6MP in the maintenance therapy for ALL. Using this system (in a large series of BCP‐ALL and T‐ALL cell lines), we verified the association of both wild‐type *NUDT15* genotype and *NT5C2* and *PRPS1* mutations with a resistance to 6MP at concentrations pharmacologically relevant to the clinical setting. Although these notions were obtained in the study of cell lines, our observations validate the clinical benefit of 6MP dose adjustment for BCP‐ALL and T‐ALL patients who carry the *NUDT15* variant allele by reducing myelosuppression without compromising anti‐leukemic efficacy. More generally, the present study provides direct evidence supporting the principle that both inherited genotypes and somatically acquired mutations are crucially implicated in the drug sensitivity of cancer cells.

## CONFLICT OF INTEREST

The authors declare no competing financial interests.

## AUTHOR CONTRIBUTIONS

**Shinpei Somazu:** Data curation (equal); Formal analysis (equal); Investigation (equal); Project administration (equal); Visualization (equal); Writing‐original draft (equal); Writing‐review & editing (equal). **Yoichi Tanaka:** Data curation (equal); Formal analysis (equal); Funding acquisition (equal); Investigation (equal); Methodology (equal); Visualization (equal); Writing‐original draft (equal); Writing‐review & editing (equal). **Minori Tamai:** Formal analysis (equal); Writing‐review & editing (equal). **Atsushi Watanabe:** Formal analysis (equal); Visualization (equal); Writing‐review & editing (equal). **Keiko Kagami:** Data curation (equal); Formal analysis (equal); Writing‐review & editing (equal). **Masako Abe:** Data curation (equal); Formal analysis (equal); Writing‐review & editing (equal). **Daisuke Harama:** Formal analysis (equal); Writing‐review & editing (equal). **Tamao Shinohara:** Formal analysis (equal); Writing‐review & editing (equal). **Koshi Akahane:** Formal analysis (equal); Writing‐review & editing (equal). **Kumiko Goi:** Formal analysis (equal); Writing‐review & editing (equal). **Kanji Sugita:** Resources (equal); Supervision (equal); Writing‐review & editing (equal). **Takaya Moriyama:** Conceptualization (equal); Methodology (equal); Writing‐review & editing (equal). **Jun Yang:** Conceptualization (equal); Methodology (equal); Writing‐review & editing (equal). **Hiroaki Goto:** Resources (equal); Writing‐review & editing (equal). **Masayoshi Minegishi:** Resources (equal); Writing‐review & editing (equal). **Shotaro Iwamoto:** Resources (equal); Writing‐review & editing (equal). **Junko Takita:** Resources (equal); Writing‐review & editing (equal). **Takeshi Inukai:** Conceptualization (equal); Data curation (equal); Funding acquisition (equal); Project administration (equal); Supervision (equal); Visualization (equal); Writing‐original draft (equal); Writing‐review & editing (equal).

## Supporting information

Supplementary Material

## Data Availability

Data openly available in a public repository that issues datasets with DOIs.
