# Supplementary material for: NUDT15 polymorphism and NT5C2 and PRPS1 mutations influence thiopurine sensitivity in acute lymphoblastic leukaemia cells
Source: J Cell Mol Med. 2021 Oct 12;25(22):10521–33. doi: 10.1111/jcmm.16981 (PMC8581340; doi:10.1111/jcmm.16981)
Supplement: Supplementary file 1 — Supplementary Material [file JCMM-25-10521-s001.pdf]

Supplemental Figure 1. Schematic representation of five haplotypes (*1 to *5) of four coding variants in *NUDT15*.

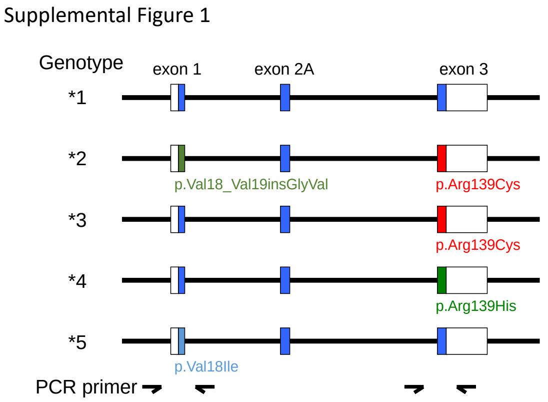

Supplemental Figure 2. Association of types of fusion with the log IC50 (day 7) values of 6MP in the 83 BCP-ALL cell lines. Gray circles indicate *BCR/ABL1*-like cell lines. P-values in Mann-Whitney analysis are indicated, when significant (p<0.05).

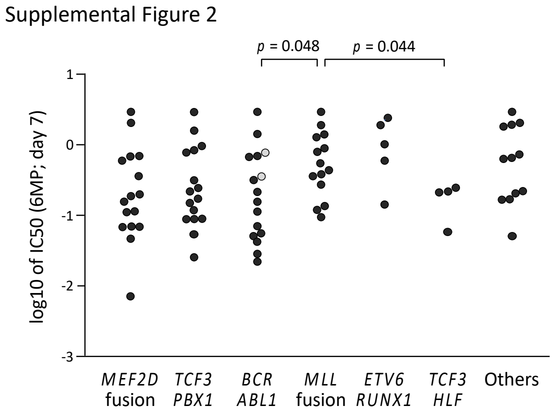

Supplemental Figure 3. Comparison of the log IC50 (day 7) values of 6MP between 29 BCP-ALL cell lines established at diagnosis and 46 BCP-ALL cell lines established at relapse. P-value in Mann-Whitney analysis is indicated, when significant (p<0.05).

Supplemental Figure 3

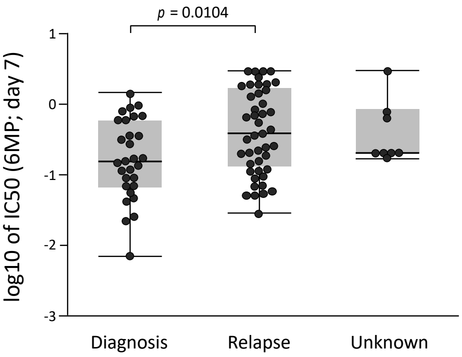

Supplemental Figure 4. Sequence of genomic PCR products of exon 1 in representative cell lines.

Supplemental Figure 4

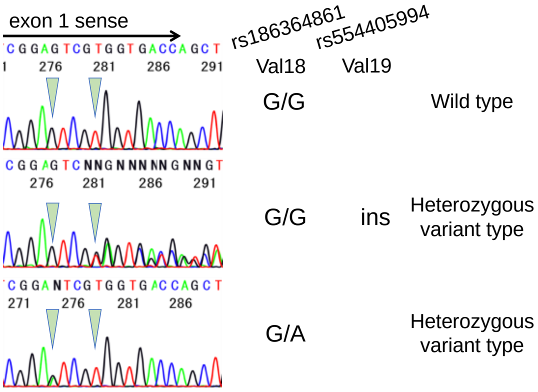

Supplemental Figure 5. Sequence of genomic PCR products of exon 3 in representative cell lines.

Supplemental Figure 5

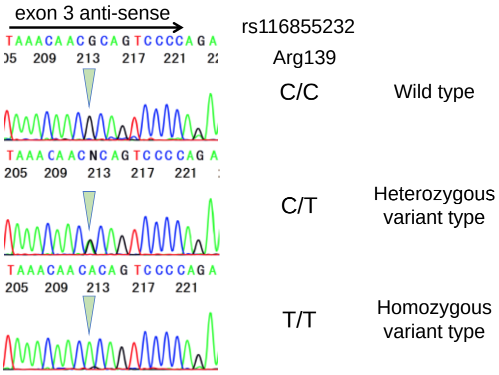

Supplemental Figure 6. Sequence of *NT5C2* mutations in four cell lines.

Supplemental Figure 6

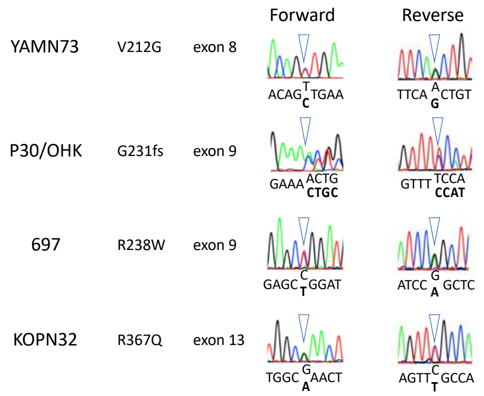

Supplemental Figure 7. Sequence of *PRPS1* mutation in KOPN72bi.

Supplemental Figure 7

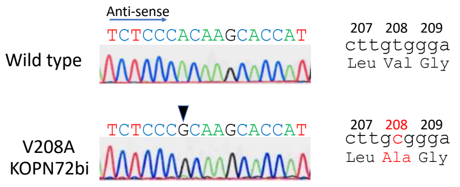

Supplemental Figure 8. Association of *NUDT15* genotype with the log IC50 (day 3) values of 6MP (left) and 6TG (right) in the 84 BCP-ALL cell lines. P-value in Mann-Whitney analysis is indicated.

Supplemental Figure 8

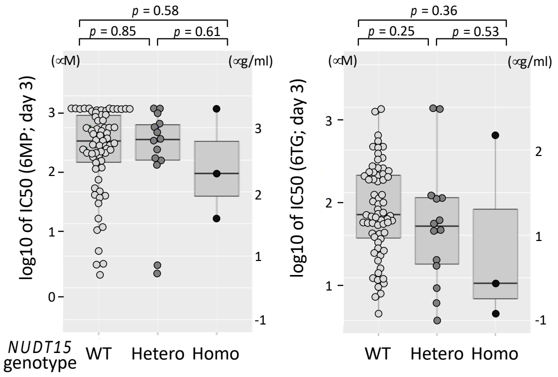

Supplemental Figure 9. Association of *NT5C2* (open circles) and *PRPS1* (open rectangle) mutations with the log IC50 (day 3) values of 6MP (left) and 6TG (right) in the 83 BCP-ALL cell

lines. P-value in Mann-Whitney analysis is indicated.

Supplemental Figure 9

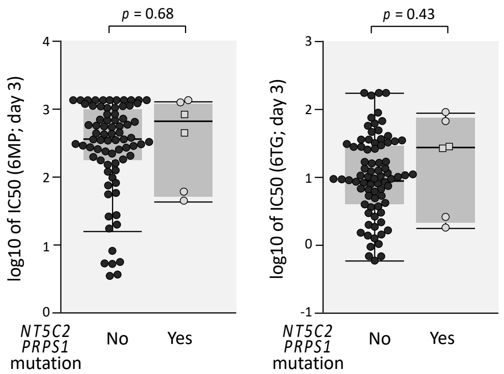

Supplemental Figure 10. Association of *NUDT15* genotype (left) and *NT5C2* and *PRPS1* mutations (right) with log IC50 (day 7) values of 6MP in the 46 BCP-ALL cell lines established at relapse. P-value in Mann-Whitney analysis is indicated.

Supplemental Figure 10

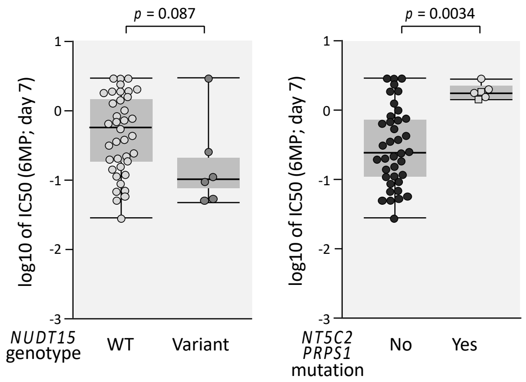

Supplemental Figure 11. Comparison of the log IC50 (day 7) values of 6MP between 35 ALL cell lines established at diagnosis and 63 ALL cell lines established at relapse. P-value in Mann-Whitney analysis is indicated, when significant (p<0.05).

Supplemental Figure 11

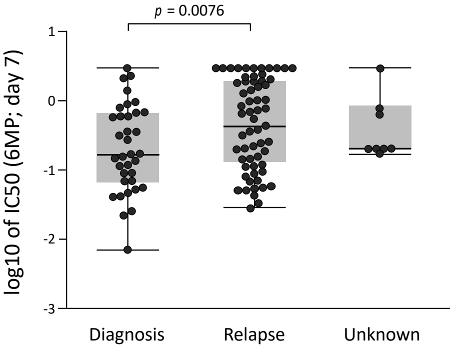

Supplemental Figure 12. Association of *NUDT15* genotype (left) and *NT5C2* and *PRPS1* mutations (right) with log IC50 (day 7) values of 6MP in the 63 ALL cell lines established at relapse. P-value in Mann-Whitney analysis is indicated.

Supplemental Figure 12

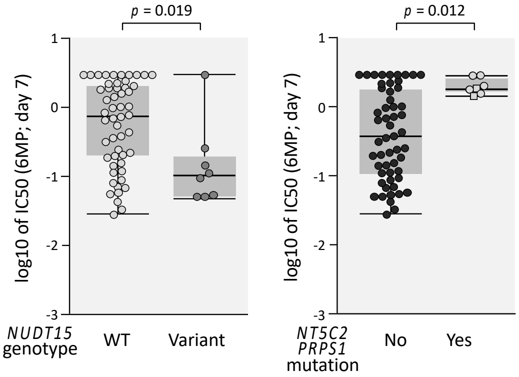

Supplemental Figure 13. Association between % mutated sequence and IC50 (day 7) values of 6MP in the 7 ALL cell lines with *NT5C2* and *PRPS1* mutations.

Supplemental Figure 13

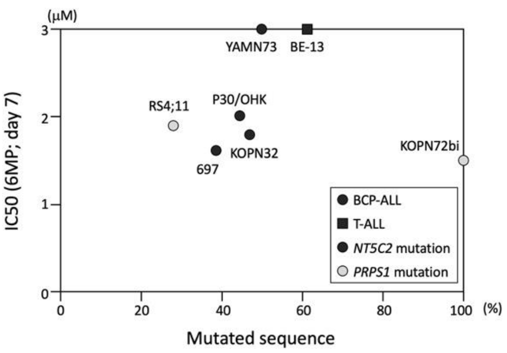

Supplemental Table 1. List of BCP-ALL cell line

Cell line	Japanese	Childhood age<16	Establishment	Fusion	*NUDT15* genotype	*NT5C2* mutation	*PRPS1* mutation
YAMN73	Yes	Yes	Relapse	*BCR-ABL1*	1*/1*	V212G	No
KOPN72bi	Yes	Yes	Relapse	*BCR-ABL1*	1*/1*	No	V208A
SK9	Yes	No	Relapse	*BCR-ABL1*	1*/1*	No	No
KOPN66bi	Yes	Yes	Relapse	*BCR-ABL1*	1*/1*	No	No
KOPN30bi	Yes	Yes	Relapse	*BCR-ABL1*	1*/1*	No	No
KOPN83bi	Yes	Yes	Relapse	*BCR-ABL1*	1*/1*	No	No
KOPN56	Yes	Yes	Relapse	*BCR-ABL1*	1*/1*	No	No
SU-Ph2	Yes	No	Relapse	*BCR-ABL1*	1*/1*	No	No
KOPN55bi	Yes	Yes	Relapse	*BCR-ABL1*	1*/1*	No	No
KOPN57bi	Yes	Yes	Diagnosis	*BCR-ABL1*	1*/3*	No	No
KCB1	Yes	Yes	Diagnosis	*BCR-ABL1*	1*/2*	No	No
YAMN91	Yes	Yes	Diagnosis	*BCR-ABL1*	1*/1*	No	No
NALM27	Yes	No	Diagnosis	*BCR-ABL1*	1*/1*	No	No
TCCY	Yes	No	NA	*BCR-ABL1*	1*/3*	No	No
KOPN49	Yes	Yes	Relapse	*BCR-ABL1* -like	1*/1*	No	No
YCUB5	Yes	Yes	Diagnosis	*BCR-ABL1* -like	1*/1*	No	No
KOCL77	Yes	Yes	Relapse	*MLL* -fusion	1*/2*	No	No
KOPN35	Yes	Yes	Relapse	*MLL* -fusion	1*/3*	No	No
KOCL44	Yes	Yes	Relapse	*MLL* -fusion	1*/1*	No	No
KOCL50	Yes	Yes	Relapse	*MLL* -fusion	1*/1*	No	No
RS4;11	No	No	Relapse	*MLL* -fusion	1*/1*	No	V289A
KOPB26	Yes	Yes	Relapse	*MLL* -fusion	1*/1*	No	No
KOCL69	Yes	Yes	Relapse	*MLL* -fusion	1*/1*	No	No
KOCL45	Yes	Yes	Relapse	*MLL* -fusion	1*/1*	No	No
KOPN1	Yes	Yes	Relapse	*MLL* -fusion	1*/1*	No	No
THP8	Yes	Yes	Diagnosis	*MLL* -fusion	1*/1*	No	No
KOCL58	Yes	Yes	Diagnosis	*MLL* -fusion	1*/1*	No	No
KOCL51	Yes	Yes	Diagnosis	*MLL* -fusion	1*/1*	No	No
YACL95	Yes	Yes	Diagnosis	*MLL* -fusion	1*/1*	No	No
KOCL33	Yes	Yes	Diagnosis	*MLL* -fusion	1*/1*	No	No
KOPN-K	Yes	Yes	Relapse	*TCF3-PBX1*	1*/3*	No	No
YAMN90R	Yes	Yes	Relapse	*TCF3-PBX1*	1*/3*	No	No
RCH	No	Yes	Relapse	*TCF3-PBX1*	1*/1*	No	No
697	No	Yes	Relapse	*TCF3-PBX1*	1*/1*	R238W	No
KOPN63	Yes	Yes	Relapse	*TCF3-PBX1*	3*/3*	No	No
KOPN54	Yes	Yes	Relapse	*TCF3-PBX1*	1*/1*	No	No
KOPN36	Yes	Yes	Relapse	*TCF3-PBX1*	1*/1*	No	No
KOPN34	Yes	No	Diagnosis	*TCF3-PBX1*	1*/1*	No	No
YCUB 6	Yes	Yes	Diagnosis	*TCF3-PBX1*	1*/1*	No	No
THP4	Yes	Yes	Diagnosis	*TCF3-PBX1*	1*/2*	No	No
YAMN92	Yes	Yes	Diagnosis	*TCF3-PBX1*	1*/1*	No	No
KOPN60	Yes	Yes	Diagnosis	*TCF3-PBX1*	1*/1*	No	No
YCUB 8	Yes	Yes	Diagnosis	*TCF3-PBX1*	1*/1*	No	No
PreALP	No	Yes	Diagnosis	*TCF3-PBX1*	1*/1*	No	No
Kasumi2	Yes	Yes	Relapse	*TCF3-PBX1*	1*/1*	No	No
SCMC-L1	Yes	Yes	NA	*TCF3-PBX1*	1*/3*	No	No
YCUB2	Yes	Yes	Relapse	*TCF3-HLF*	1*/1*	No	No
Endokun	Yes	Yes	NA	*TCF3-HLF*	1*/3*	No	No
UOCB1	No	Yes	Relapse	*TCF3-HLF*	1*/1*	No	No
HALO1	Yes	No	NA	*TCF3-HLF*	1*/3*	No	No
KOPN68	Yes	Yes	Relapse	*ETV6-RUNX1*	1*/1*	No	No
KOPN87	Yes	Yes	Relapse	*ETV6-RUNX1*	1*/1*	No	No
Reh	No	Yes	Relapse	*ETV6-RUNX1*	1*/1*	No	No
KOPN79	Yes	Yes	Relapse	*ETV6-RUNX1*	1*/1*	No	No
KOPN41	Yes	Yes	Diagnosis	*ETV6-RUNX1*	1*/1*	No	No
P30/OHK	Yes	Yes	Relapse	*MEF2D* -fusion	1*/1*	G231fs	No
KOPN70	Yes	Yes	Relapse	*MEF2D* -fusion	1*/1*	No	No
YCUB7	Yes	Yes	Relapse	*MEF2D* -fusion	1*/1*	No	No
L-KUM	Yes	Yes	Relapse	*MEF2D* -fusion	1*/1*	No	No
KOS20	Yes	Yes	Relapse	*MEF2D* -fusion	1*/1*	No	No
YAMN74	Yes	Yes	Relapse	*MEF2D* -fusion	1*/1*	No	No
KOPN61	Yes	Yes	Diagnosis	*MEF2D* -fusion	1*/1*	No	No
THP5	Yes	Yes	Diagnosis	*MEF2D* -fusion	3*/3*	No	No
YAMN96	Yes	Yes	Diagnosis	*MEF2D* -fusion	1*/5*	No	No
KOPN39	Yes	Yes	Diagnosis	*MEF2D* -fusion	1*/3*	No	No
YCUB4	Yes	Yes	Diagnosis	*MEF2D* -fusion	1*/1*	No	No
L-ASK	Yes	Yes	Diagnosis	*MEF2D* -fusion	1*/1*	No	No
THP7	Yes	Yes	Diagnosis	*MEF2D* -fusion	1*/1*	No	No
KOPN71	Yes	Yes	Diagnosis	*MEF2D* -fusion	1*/1*	No	No
KOPN46	Yes	Yes	Diagnosis	*MEF2D* -fusion	1*/1*	No	No
MBMY	Yes	Yes	Diagnosis	*MEF2D* -fusion	1*/1*	No	No
KOPN62	Yes	No	NA	*MEF2D* -fusion	1*/1*	No	No
KOPN32	Yes	Yes	Relapse	Others	1*/1*	R367Q	No
KOPN85	Yes	Yes	Relapse	Others	1*/1*	No	No
KOPN84	Yes	Yes	Relapse	Others	1*/2*	No	No
KOPN40	Yes	Yes	Relapse	Others	1*/1*	No	No
KCB4	Yes	Yes	Relapse	Others	1*/1*	No	No
MBKG	Yes	Yes	Relapse	Others	1*/1*	No	No
MBOK	Yes	Yes	Relapse	Others	1*/1*	No	No
Nalm6	No	No	Relapse	Others	1*/1*	No	No
KOPN75	Yes	Yes	Diagnosis	Others	1*/1*	No	No
SCMCL2	Yes	Yes	NA	Others	3*/3*	No	No
KOPB38	Yes	Yes	NA	Others	1*/1*	No	No
KOPB59	Yes	Yes	NA	Others	1*/1*	No	No

Supplemental Table 2. List of T-ALL cell line

Cell line	Japanese	Childhood age<16	Establishment	Fusion	*NUDT15* genotype	*TPMT* rs1142345	*NT5C2* mutation	*PRPS1* mutation
DND-41	NA	Yes	Diagnosis	*BCL11B-TLX3*	1*/1*	WT	No	No
HPB-ALL	Yes	Yes	Diagnosis	*BCL11B-TLX3*	1*/1*	WT	No	No
KOPT-K1	Yes	Yes	Relapse	*LMO2-TCRG*	1*/1*	WT	No	No
SUP-T13	No	Yes	Relapse	*MLL-ENL*	1*/1*	WT	No	No
SKW-3	NA	No	Diagnosis	*MYC-TCRA*	1*/1*	WT	No	No
Kasumi5	Yes	No	Relapse	*NUP98-RAP1GDS1*	1*/1*	WT	No	No
LOUCY	No	No	Relapse	*SET-NUP214*	1*/1*	WT	No	No
CCRF-CEM	No	Yes	Relapse	*STIL-TAL1*	1*/1*	WT	No	No
HSB2	No	Yes	Relapse	*STIL-TAL1*	1*/1*	WT	No	No
L-KAW	Yes	Yes	Relapse	*STIL-TAL1*	1*/3*	WT	No	No
MOLT-16	NA	Yes	Relapse	*STIL-TAL1*	1*/1*	WT	No	No
PF382	No	Yes	Relapse	*STIL-TAL1*	1*/1*	WT	No	No
RPMI-8402	No	No	Diagnosis	*STIL-TAL1*	1*/1*	WT	No	No
ALL-SIL	No	No	Relapse	*TLX1-TCRD*	1*/1*	WT	No	No
BE-13	No	Yes	Relapse	Others	1*/1*	WT	R29Q	No
JURKAT	No	Yes	Relapse	Others	1*/1*	WT	No	No
KOPT-5	Yes	Yes	Relapse	Others	1*/1*	WT	No	No
L-MAT	Yes	Yes	Diagnosis	Others	1*/1*	A/G	No	No
MOLT-13	NA	Yes	Relapse	Others	1*/1*	WT	No	No
MOLT-4	NA	No	Relapse	Others	1*/1*	WT	No	No
P12/ICK	Yes	Yes	Diagnosis	Others	1*/1*	WT	No	No
PEER	No	Yes	Relapse	Others	1*/1*	WT	No	No
TALL-1	Yes	No	Relapse	Others	1*/3*	WT	No	No

Supplemental Table 3. List of *NT5C2* and *PRPS1* mutations

Cell line	Lineage	Gene	Mutation	Amino acid change	Frequency (%)
BE-13	T	*NT5C2*	c.86G>A	p.R29Q	60.2
YAMN73	BCP	*NT5C2*	c.656T>C	p.V212G	49.8
P32/OHK	BCP	*NT5C2*	c.693del	p.K231Nfs	44.9
697	BCP	*NT5C2*	c.712C>T	p.R238W	38.8
KOPN32	BCP	*NT5C2*	c.1100G>A	p.R367Q	46.6
KOPN72bi	BCP	*PRPS1*	c.623T>C	p.V208A	100
RS4;11	BCP	*PRPS1*	c.866T>C	p.V289A	28.1
